# Whole genome sequencing and comparative genomic analyses of *Planococcus alpniumensis* MSAK28401^T^, a new species isolated from Antarctic krill

**DOI:** 10.1186/s12866-021-02347-3

**Published:** 2021-10-22

**Authors:** Yuanyuan Wang, Lingbo Ma, Jian He, Zixuan Liu, Shaoping Weng, Lumin Wang, Jianguo He, Changjun Guo

**Affiliations:** 1 State Key Laboratory for Biocontrol / Southern Laboratory of Ocean Science and Engineering (Guangdong, Zhuhai), School of Marine Sciences, Sun Yat-sen University, 135 Xingang Road West, Guangzhou, 510275 People’s Republic of China; 2 Guangdong Province Key Laboratory for Aquatic Economic Animals, and Guangdong Provincial Key Laboratory of Marine Resources and Coastal Engineering, Sun Yat-sen University, 135 Xingang Road West, Guangzhou, 510275 People’s Republic of China; 3 Key Laboratory of the East China Sea and Oceanic Fishery Resources Exploitation, Ministry of Agriculture, East China Sea Fisheries Research Institute, Shanghai, 116023 People’s Republic of China

**Keywords:** *Planococcus*, Antarctic krill, Genomic analysis, Extremophiles

## Abstract

**Background:**

Extremophiles have attracted much attention in the last few decades, as they possess different properties by producing certain useful metabolites. However, the secondary metabolism of the extremophiles of Antarctic krill has received little attention.

**Results:**

In this study, a new bacterial strain MSAK28401^T^ from Antarctic krill was isolated and identified. The results of analysis on phenotypic, chemotaxonomic, and genomic characteristics showed that the strain MSAK28401^T^ belongs to the genus *Planococcus*. Cells of this strain were coccoid (0.89–1.05 μm) and aerobic. The majority of the fatty acid content was C_15:0_ anteiso (37.67 ± 0.90%) followed by C_16:1_ ω7c alcohol (10.37 ± 1.22%) and C_16:0_ iso (9.36 ± 0.71%). The calculated average nucleotide identity and DNA–DNA hybridization values between the strain MSAK28401^T^ and type strains *P. citreus* DSM 20549^T^ and *P. rifietoensis* M8^T^ were lower than 91 and 70%, respectively. The strain MSAK28401^T^ (=KCTC 43283^T^ and MCCC 1k05448^T^) represented a new member of the genus *Planococcus* and was named *P. alpniumensis* sp. nov. Moreover, genes involved in the degradation of aromatic compounds (e.g., salicylate, gentisate, and quinate) were found in the genome, implying that strain MSAK28401^T^ has an aromatic compound as its potential metabolite. This work will help us understand the genomic characteristics and potential metabolic pathway of *Planococcus* from Antarctic krill.

**Conclusions:**

This study reported the genomic information and phenotypic characteristics of the new strain *P. alpniumensis* MSAK28401^T^ isolated from Antarctic krill, and provided the genome information of *Planococcus* strains for further studying the function roles in aromatic compound metabolism.

**Supplementary Information:**

The online version contains supplementary material available at 10.1186/s12866-021-02347-3.

## Background

The genera *Planococcus* was initially found and proposed by Migula and has been continuously revised [1]. It was classified as *Planococcaceae* of *Firmicutes*, and 30 species had been published to date. Recently, the species *P. okeanokoites* and *P. mcmeekinii* [2], *P. psychrophilum* [3], *P. stackebrandtii*, and *P. alkanoclasticum* [4, 5] were reclassified to the genera *Planomicrobium* based on phenotypic properties, G + C content in DNA, fatty acid composition, and menaquinone profiles. *P. halophilus* was classified under the genera *Marinococcus* [6, 7]. These changes indicated that genus *Planococcus* and *Planomicrobium* have a close phylogenetic relationship. Usually, the main splitting points of the 16S rRNA sequence between the genera *Planococcus* and *Planomicrobium* were located at sites 183 and 190 (*E. coli* counting), which in the *Planococcus* are T and A, whereas in the *Planomicrobium* are C and G [8].

*Planococcus* has the following known features: Gram-positive, multicellular morphology (cocci, short rod, or rod), aerobic, and no sporulation [8]. Representative strains of genus *Planococcus* usually grow in cold and/or saline-alkali soil with high salt concentrations, e.g., Arctic, Antarctic, and marine environments [9–11]. *Planococcus* has attracted much attention, because they can produce carotenoids of biotechnological significance; this metabolite has potential applications as the ingredient of cosmetics, food or feed additives, and antioxidants [12]. *Planococcus* can also degrade and process various contaminants, such as heavy metals and phenols, and play an important role in the bioremediation of extreme environments [13, 14].

In the present study, a new strain *P. alpniumensis* MSAK28401^T^ of the genus *Planococcus* from Antarctic krill was isolated and identified using taxonomic, phylogenetic, chemotaxonomic, whole-genomic, and comparative genomic analysis.

## Results

### Isolation, identification, and phylogenetic analysis

**Fig. 1:**
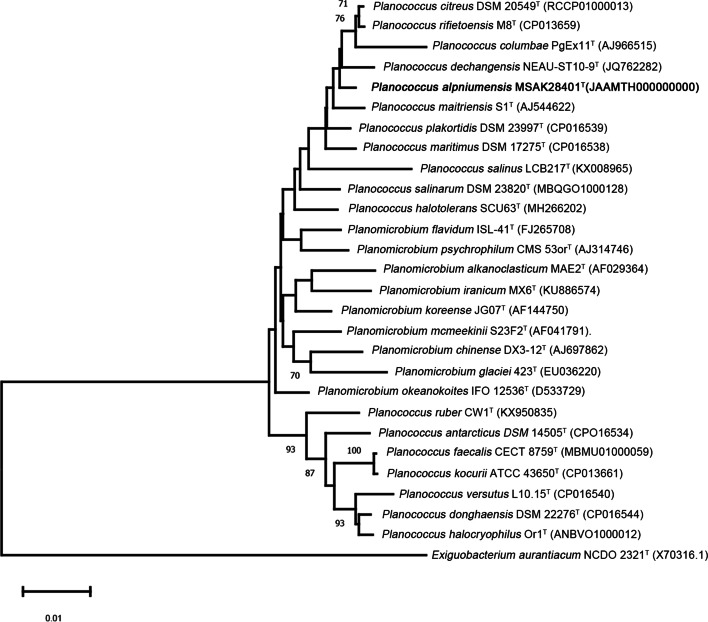
The NJ tree shows *Planococcus* sp. at the position of concerned taxa on the 16S rRNA gene. Bootstrap values of ≥70% were shown at nodes

The single-bacterial MSAK28401^T^ was obtained by mixing culture on Luria-Bertani (LB) agar. The 16S rRNA sequence alignment against GenBank revealed that the strain MSAK28401^T^ belonged to the genus *Planococcus*, and it showed 98.62, 98.55, 98.43, 98.20, and 97.79% similarity with the corresponding gene sequences of *P. citreus* DSM20549^T^, *P. rifietornsis* M8^T^, *P. maitriensis* S1^T^, *P. dechangensis* NEAU-ST10-9^T^, and *P. maritimus* DSM17275^T^, respectively (additional File 1: Table S1). The 16S rRNA phylogenetic tree showed that strain MSAK28401^T^ was clustered with four species of the genus *Planococcus,* and placed in an independent branch (Fig. 1). These results suggested that strain MSAK28401^T^ belongs to the genus *Planococcus*.

### Phenotypic characterization

**Fig. 2:**
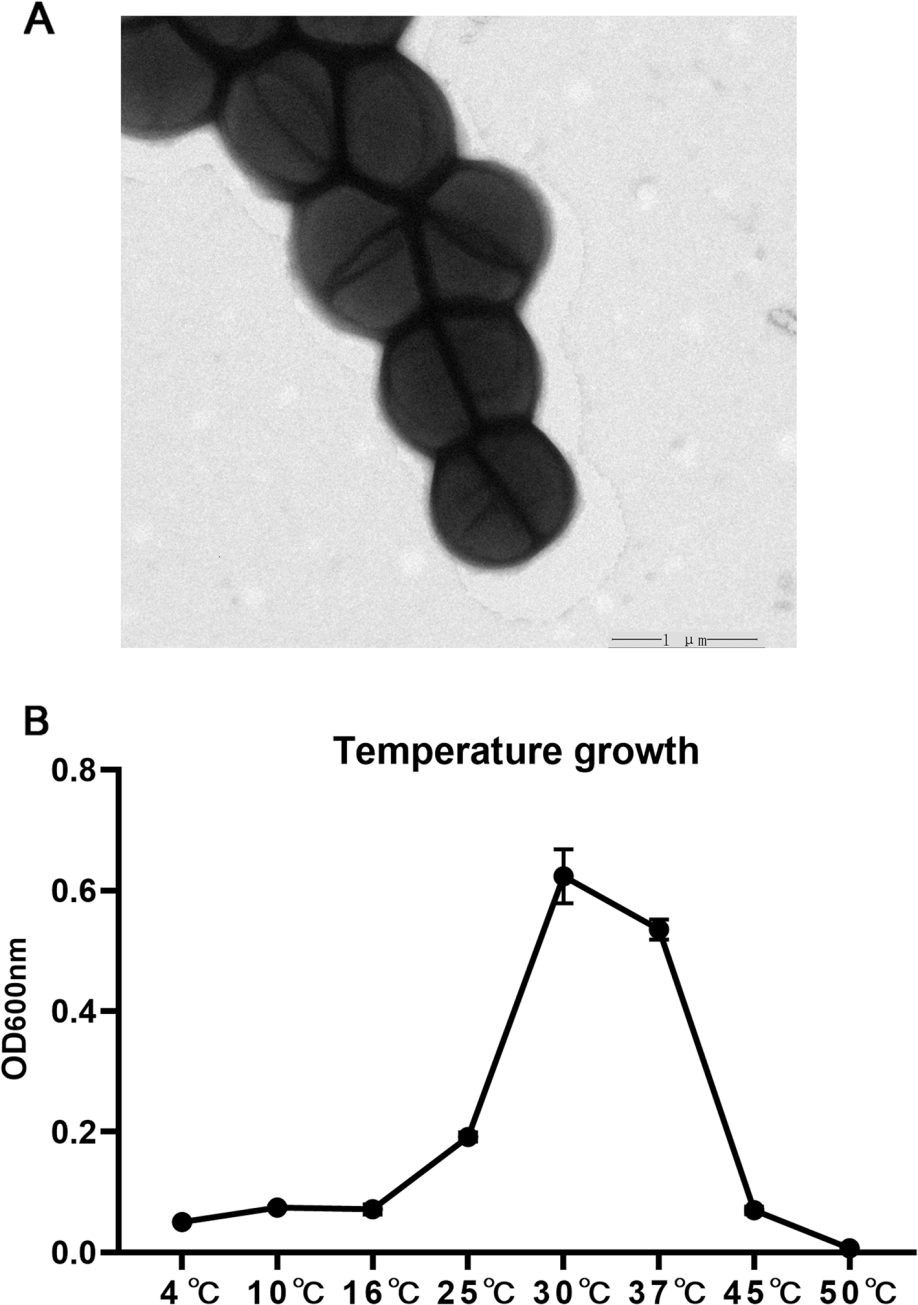
**a** Transmission electron micrograph showing exponentially growing cells of strain MSAK28401^T^. Bar, 1 μm. **b** Temperature growth curve of isolates. The abscissa is different temperatures, the ordinate is the absorbance value of OD_600nm_

The transmission electron microscopy observations showed that cell coccoid and the diameter of strain MSAK28401^T^ was 0.89–1.05 μm with a thick cell wall (Fig. 2A). The isolates could grow in the range of 4–50 °C, and the optimal growth temperature was 30 °C (Fig. 2B). The phenotypic characteristics of strain MSAK28401^T^ and related species as shown in Table 1. Strain MSAK28401^T^ differed from the type strains of *P. citreus* DSM20549^T^, *P. rifietornsis* M8^T^, and *P. maitriensis* S1^T^ in the assimilation of *β*-methyl-_D_-glucoside, _D_-aspartic acid, _L_-arginine, quinic acid, _D_-glucuronic acid, and _L_-malic acid. Strain MSAK28401^T^ was distinguished from other species of the genus *Planococcus* by using some carbon sources and by producing acids from certain sugars. Phenotypic characteristics suggested that the strain MSAK28401^T^ may represent a new *Planococcus* species and was named *P. alpniumensis* sp. nov.

**Table 1:** Differential phenotypic characteristics of *Planococcus* sp. and closely related *Planococcus* species

Characteristic	1	2	3	4^a,b^
**Carbon source utilization:**				
Dextrin	–	+	+	-^a^
D-Cellobiose	–	–	+	-^a^
D-Turanose	+	+	+	NR
α-D-Lactose	–	+	+	-^a^
D-Melibiose	–	+	–	+^a^
β-Methyl-D-Glucoside	–	+	+	NR
α-D-Glucose	–	–	+	+^a^
D-Mannose	+	+	+	-^a^
D-Fructose	–	+	+	+^a^
D-Galactose	+	+	+	-^a^
3-Methyl Glucose	+	+	+	NR
D-Sorbitol	+	+	+	NR
D-Mannitol	+	+	+	-^a^
D-Arabitol	–	+	–	NR
D-Glucose-6-PO4	–	+	–	NR
D-Aspartic Acid	–	+	+	+^b^
L-Alanine	–	+	+	-^a^
L-Arginine	–	+	+	+^a^
L-Aspartic Acid	–	–	+	NR
L-Glutamic Acid	–	+	+	-^a^
L-Pyroglutamic Acid	–	+	–	NR
D-Galacturonic Acid	–	+	–	+^b^
L-Galactonic Acid Lactone	–	+	+	NR
D-Gluconic Acid	+	+	+	NR
D-Glucuronic Acid	–	+	+	NR
Glucuronamid	–	+	+	-^b^
Quinic Acid	–	+	+	+^b^
D-Lactic Acid Methyl Ester	+	+	+	-^a^
L-Malic Acid	–	+	+	NR
Tween 40	+	+	+	-^b^
β-Hydroxy-D,L Butyric Acid	–	+	–	NR
α-Keto-Butyric Acid	–	+	–	NR
Acetoacetic Acid	+	+	+	NR
Propionic Acid	–	–	+	-^b^
Acetic Acid	+	+	+	NR
**Chemical sensitivity:**				
1% NaCl	+	+	+	+^a^
4% NaCl	+	+	–	+^a^
8% NaCl	–	+	–	+^a^
Tetrazolium Violet	+	–	–	NR
Nalidixic Acid	–	+	–	-^a^
Lithium Chloride	+	+	+	NR
Aztreonam	+	+	+	NR

1:MSAK28401^T^ (data from this study), 2:*P. citreus* DSM 20549^T^, 3: *P. rifietoensis* M8^T^, 4: *P. maitriensis* S1^T^. (Alam et al., 2003; Suresh et al. 2007; Gan et al., 2018). +, Present; −, absent, NR, not reported

### Fatty acid analysis

The details of the fatty acid profiles of the strain MSAK28401^T^ and three related species of *P. citreus* DSM 20549^T^, *P. rifietoensis* M8^T^, and *P. maitriensis* S1^T^were described (Table 2). These major fatty acids (> 5%) of strain MSAK28401^T^ were C_15:0_ anteiso (37.67 ± 0.90%), C_16:1_ ω7c alcohol (10.37 ± 1.22%), and C_16:0_ iso (9.36 ± 0.71%). The main fatty acid with the highest content is C_15:0_ anteiso. The other major fatty acids that were the most abundant in strain MSAK28401^T^, namely, C16:0 iso (9.36 ± 0.71%), C_16:1_ ω7c alcohol (10.37 ± 1.22%), and C_14:0_ iso (7.80 ± 0.15%), showed quantitative differences with those in the two related type species. Results of comparing fatty acid types and proportions suggested that the strain MSAK28401^T^ can be distinguished from the two species of a cluster in the phylogeny.

**Table 2:** Cellular fatty acid composition of *Planococcus* sp. and *P. citreus* DSM 20549^T^, *P. rifietoensis* M8^T^, and *P. maitriensis* S1^T^

Fatty acids	1	2	3	4^a,b,c^
C_12:0_	1.29 ± 0.37	1.14 ± 0.57	2.57 ± 1.29	–
C_14:0_ iso	7.80 ± 0.18	1.41 ± 0.85	9.26 ± 0.17	–
C_15:0_ anteiso	37.67 ± 0.90	44.22 ± 5.39	37.28 ± 1.60	27.3 ± 2.05
C_15:0_ iso	6.44 ± 0.47	0.66 ± 0.54	3.51 ± 0.57	2.8 ± 1.62
C_16:0_	1.33 ± 0.21	7.15 ± 1.13	1.96 ± 0.63	7.2 ± 1.11
C_16:0_ iso	9.36 ± 0.71	5.89 ± 1.45	9.05 ± 3.38	9.2 ± 3.33
C_16:1_ ω11c	1.38 ± 0.07	2.35 ± 0.94	1.49 ± 0.25	–
C_16:1_ ω7c alcohol	10.37 ± 1.22	3.62 ± 0.59	9.91 ± 0.68	N
C_17:0_	2.14 ± 0.66	3.03 ± 1.04	2.14 ± 0.24	5.3 ± 1.03
C_17:0_ 10-methyl	0.35 ± 0.03	0.26 ± 0.09	0.50 ± 0.16	N
C_17:0_ anteiso	5.76 ± 0.47	14.18 ± 0.72	5.84 ± 1.05	6.6 ± 1.61
C_17:0_ iso	2.41 ± 0.29	0.53 ± 0.56	1.58 ± 0.34	N
C_17:1_ iso I/anteiso B	2.03 ± 0.34	4.31 ± 0.31	2.96 ± 2.06	N
C_17:1_ iso ω10c	1.25 ± 0.07	0.28 ± 0.00	1.16 ± 0.32	N
C_17:1_ ω9c	2.92 ± 0.15	0.64 ± 0.00	3.95 ± 1.83	N
C_18:0_	0.53 ± 0.12	4.71 ± 1.22	1.01 ± 0.93	4.0 ± 1.05
C_18:0_ iso	1.61 ± 0.02	1.46 ± 0.50	2.29 ± 0.63	3.1 ± 1.55
C_18:1_ ω9c	1.04 ± 0.36	1.13 ± 0.45	1.33 ± 0.41	4.2 ± 1.70
C_19:0_ anteiso	0.34 ± 0.07	0.89 ± 0.17	0.44 ± 0.03	N

All values< 0.5% are not shown;

All the strains were tested under the same growth conditions

1 MSAK28401^T^. 2 *P. citreus* DSM 20549^T^, 3 *P. rifietoensis* M8^T^,4 *P. maitriensis* S1^T^,

– not detected, N none data

^a^ Data from: Alam et al.(2003); ^b^ Data from:Gan et al.(2018); ^c^ Data from: Suresh Gan et al.(2007)

### Genome properties and mining

**Fig. 3:**
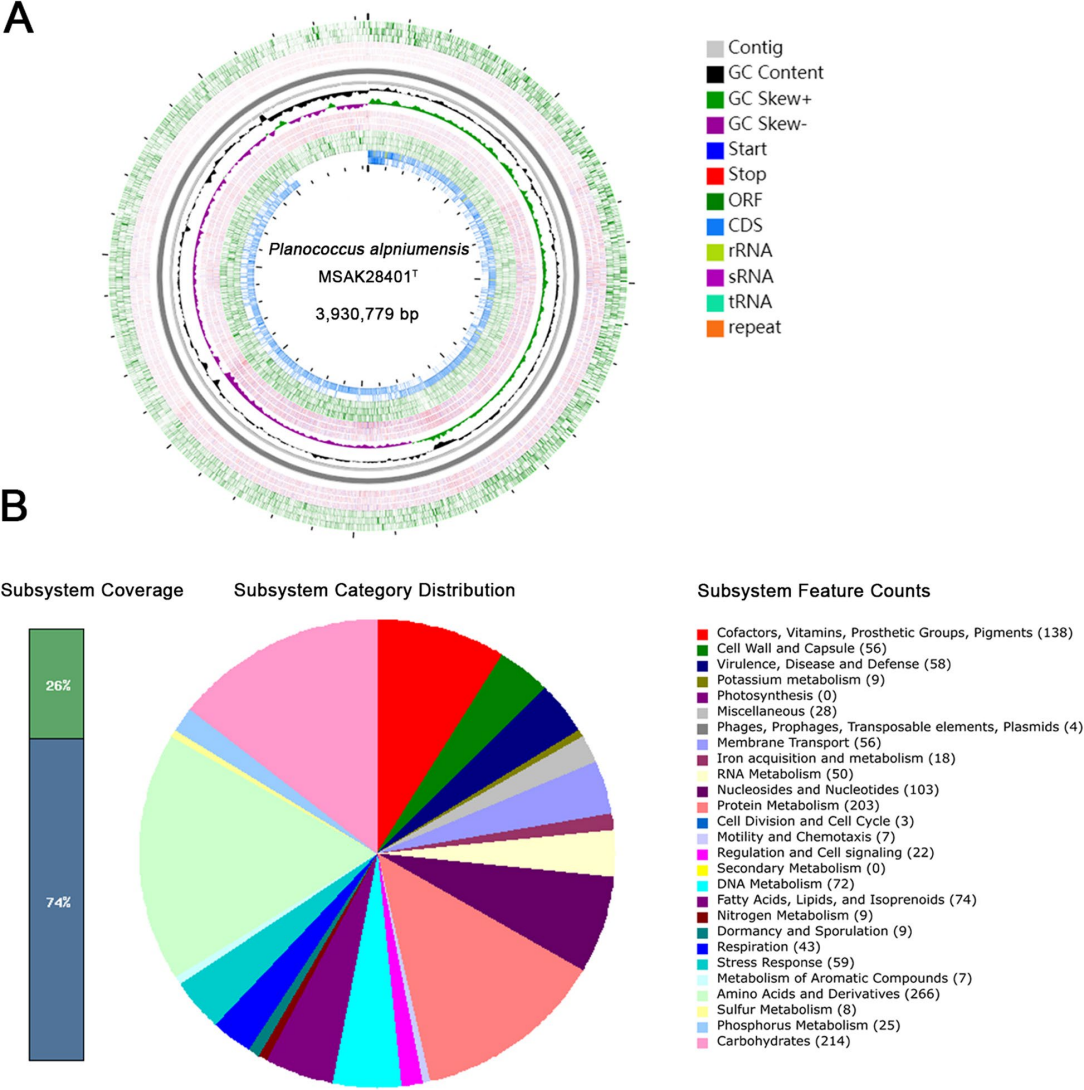
A draft map of whole-genome and distribution of annotated genes of strain *Planococcus* MSAK28401^T^ sp. **a** Genomic circle diagram of a new strain MSAK28401^T^. **b** Annotate *Planococcus* sp. genome information using RAST server

The genome of strain MSAK28401^T^ formed from 10 contigs, and the genomic length was 3,930,779 bp. The G + C content was 47.15%. We identified 3998 genes and 3897 codifying sequences (Table 3 and Fig. 3A) and assigned them to 27 subsystems with SEED viewer using the RAST pipeline (Fig. 3B and additional File 2: Table S2). Nevertheless, only 26% (1150, genes) of this genome was annotated, and the other 74% was not assigned to the RAST subsystems. The most represented subsystem features were amino acids and derivatives (266), carbohydrates (214), protein metabolism (203), cofactors, vitamins, prosthetic group, and pigments (138). Notably, several genes involved in dormancy and sporulation were also found in strain MSAK28401^T^.

**Table 3:** Genome statistics of the *Planococcus* sp.

Attribute	Value	% of total^**a**^
Genome size (bp)	3,930,779	100.00
DNA coding region (bp)	3,380,475	86.00
DNA G + C (bp)	1,631,417	47.15
DNA scaffolds	10	–
Total genes	3998	100.00
Protein coding genes	3835	95.92
RNA gene	101	2.53
Pseudo genes	62	1.55
Gene with function prediction	3258	
Genes assigned to COGs	2765	69.16
Genes assigned Pfam domains	10	0.25
Genes with signal peptides	169	4.23
Genes with transmembrane helices	1123	28.09
CRISPR repeats	219	5.45

^a^The total is based on either the size of the genome in base pairs or the total number of protein coding genes in the annotated genome

**Fig. 4:**
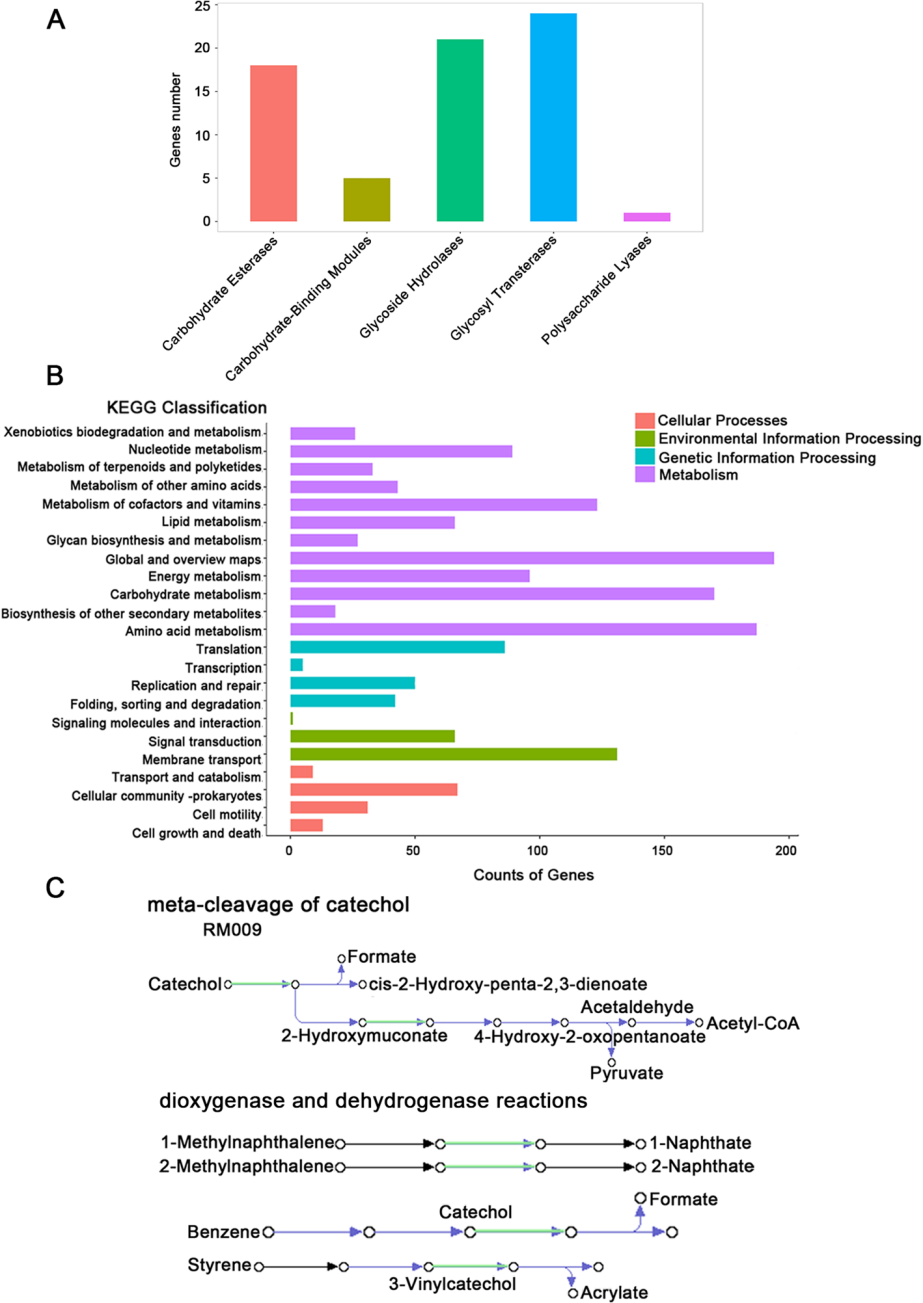
CAZy and KEGG annotation class distribution. **a** CAZy annotation classification distribution map. The abscissa is the CAZy classification, and the ordinate is the number of genes annotated to the corresponding classification. **b** KEGG annotation statistics chart at Level 2. The horizontal axis is the number of genes, the vertical axis represents the name of the Level 2 pathway, and the number on the right side of the column is the number of genes annotated to the Level 2 pathway. **c** Aromatic hydrocarbon degradation pathways involved in isolates

Carbohydrate-related enzymes and activity annotations of presumed genes showed that 24 genes encoded glycosyl transferases (GT) and 21 genes encoded glycosyl hydrolases (GH) (Fig. 4A and additional File 3: Table S3). KofamKOALA analysis results showed that almost all of the major metabolic pathways of bacteria were found in the genome of strain MSAK28401^T^ (Fig. 4B and additional File 4: Table S4). Most genes were related to amino acid and carbohydrate metabolism, suggesting that MSAK28401^T^ might possess the efficient nutrient uptake systems. In-depth analysis of the metabolic pathways of the strain MSAK28401Trevealed that genes related to aromatic hydrocarbon degradation pathways, such as catechol 2,3-dioxygenase (gene 0402), 4-oxalocrotonate tautomerase (gene 2794), and S-(hydroxymethyl) glutathione dehydrogenase / alcohol dehydrogenase (gene 2852) (Additional File 5: Table S5). Notably, 4-oxalocrotonate tautomerase (EC 5.3.2.-4-OT) is an enzyme that forms part of a bacterial metabolic pathway that oxidatively catabolizes toluene, o-xylene, 3-ethyltoluene, and 1,2,4-trimethylbenzene into intermediates of the citric acid cycle. In addition, we mapped the relevant pathways of aromatic hydrocarbons that the isolate may be involved in degradation (Fig. 4C). Above results indicated this isolate have a potential for application to the process of aromatic hydrocarbon metabolism.

### Genetic relatedness and Pan-genome analysis

**Fig. 5:**
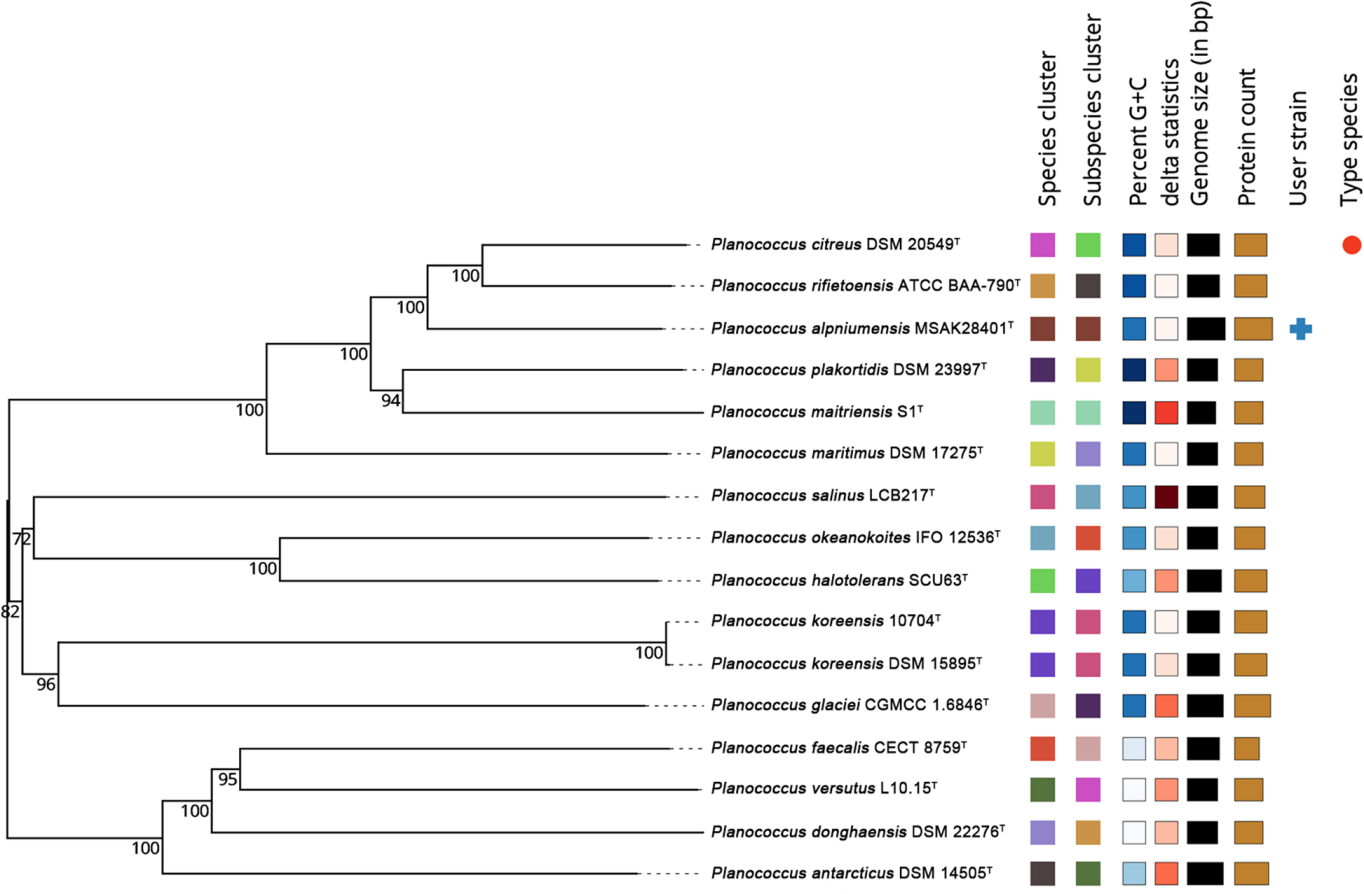
The phylogenetic tree was constructed based on the whole genome using the Genome-BLAST distance phylogenetic method (GBDP) tool. According to the GBDP distance formula d_5,_ the branch lengths were scaled

The phylogenetic tree of GBDP determined the phylogenetic position of strains, and it showed that the strain MSAK28401^T^ was clustered with *P. citreus* DSM 20549^T^ and *P. rifietoensis* M8^T^ (Fig. 5). The DDH and ANIb values between the strain MSAK28401^T^ and related species *P. citreus* DSM 20549^T^ and *P. rifietoensis* M8^T^ were less than 70 and 91%, respectively (Table 4 and Table 5), which were below the threshold for species delineation. The above results support the affiliation of the strain MSAK28401^T^ to a new species of the genus *Planococcus*.

**Table 4:** The dDDH values are provided along with their confidence intervals (C.I.) for the GBDP formula 2

Query	Subject	d4	C.I. d4
MSAK28401^T^	*Planococcus rifietoensis* ATCC BAA-790	42.8	[40.3–45.4]
MSAK28401^T^	*Planococcus citreus* DSM 20549	42.3	[39.8–44.9]
MSAK28401^T^	*Planococcus maitriensis* S1	35.7	[33.3–38.3]
MSAK28401^T^	*Planococcus plakortidis* DSM 23997	34.0	[31.5–36.5]
MSAK28401^T^	*Planococcus maritimus* DSM 17275	29.7	[27.4–32.2]

**Table 5:** Calculated ANIb values for available genomes of the type strains from the type species of the genera included in the genus *Planococcus* (the accession numbers for these genomes are in parentheses)

Species	***Planococcus sp***	DSM 14505^**T**^	DSM 22276^**T**^	DSM 20549^**T**^	DSM 24743^**T**^	ATCC 43650^**T**^	17275^**T**^	S1^**T**^	DSM 23997^**T**^	M8^**T**^	ISL-16^**T**^	PAMC 21323	L10.15^**T**^	SCU63^**T**^
*Planococcus* sp	100													
DSM 14505^T^	72.95	100												
DSM 22276^T^	72.07	79.62	100											
DSM 20549^T^	90.31	72.44	71.27	100										
DSM 24743^T^	72.04	80.17	88.82	72.01	100									
ATCC 43650^T^	72.09	81.52	80.90	72.03	81.63	100								
17275^T^	84.92	72..73	72.12	85.04	72.27	72.36	100							
S1^T^	87.86	72.30	71.07	89.22	71.11	71.25	83.85	100						
DSM 23997^T^	87.07	72.57	71.56	87.83	71.55	71.66	83.59	88.93	100					
M8^T^	90.49	72.74	71.75	92.26	71.88	72.01	84.58	88.51	87.36	100				
ISL-16^T^	72.59	73.09	71.78	72.50	71.93	72.15	72.08	72.76	72.50	72.46	100			
PAMC 21323	71.99	79.50	85.59	71.75	86.72	81.10	72.13	71.59	71.52	71.78	72.13	100		
L10.15^T^	71.77	79.70	80.76	71.53	81.16	81.85	71.91	71.32	71.14	71.68	71.89	81.25	100	
SCU63^T^	72.34	72.86	72.22	72.22	72.22	72.33	71.97	72.48	72.26	72.36	80.83	72.15	72.00	100

*Planococcus* sp. *Planococcus antarcticus* DSM 14505^T^, *Planococcus donghaensis* DSM 22276^T^, *Planococcus citreus* DSM 20549^T^, *Planococcus halocryophilus* DSM 24743^T^, *Planococcus kocurii* ATCC 43650^T^, *Planococc maritimus* 17275^T^, *Planococcus maitriensis* S1^T^, *Planococcus plakortidis* DSM 23997^T^, *Planococcus rifietoensis* M8^T^, *Planococcus salinarum* ISL-16^T^, *Planococcus sp*. PAMC 21323, *Planococcus versutus* L10.15^T^, Planococcus halotolerans SCU63^T^

**Fig. 6:**
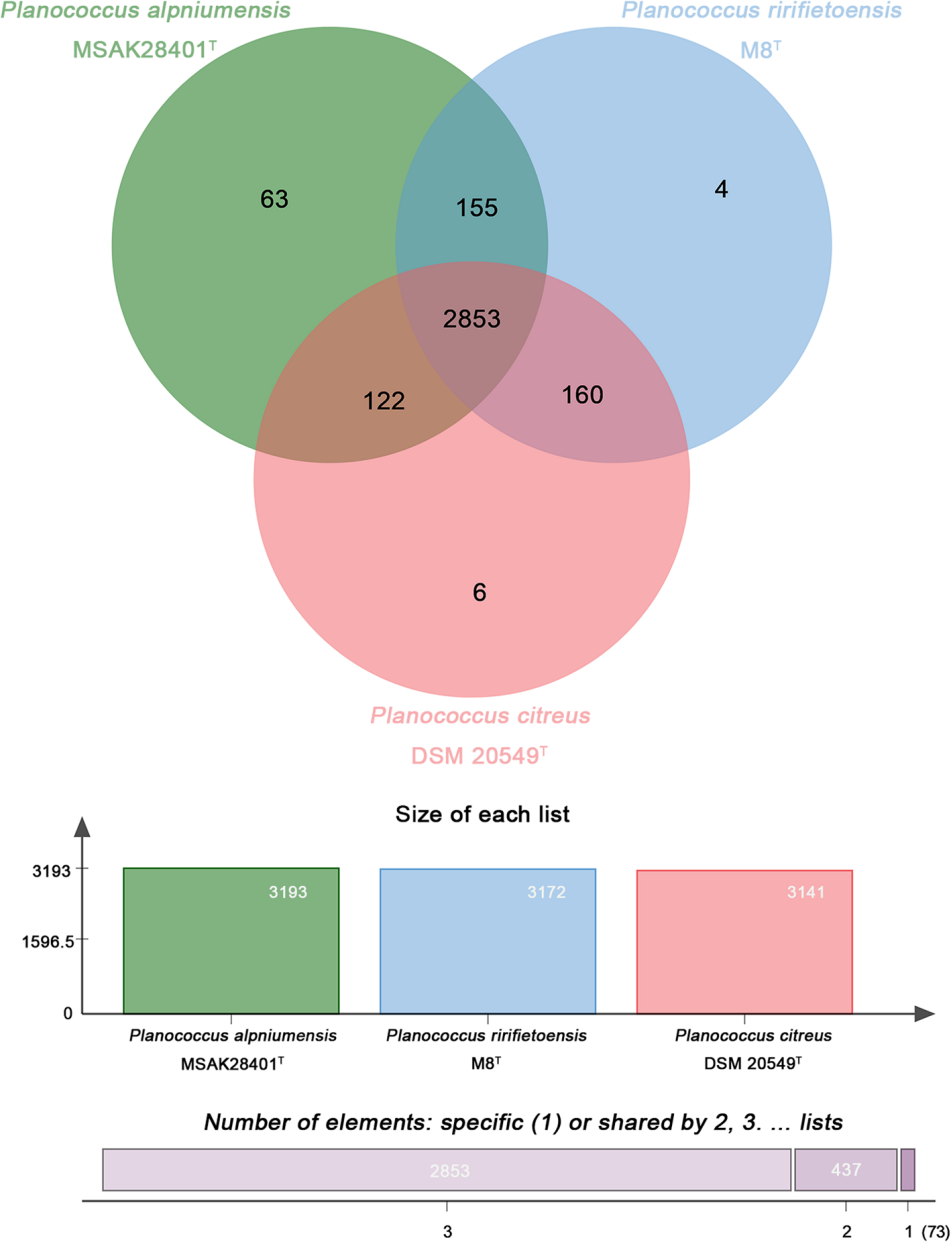
The Venn diagram and the bar graph depict the comparative genomics among the genomes of *P. alpniumensis* MSAK28401^T^, *P. citreus* DSM 20549^T^, and *P. rifietoensis* M8^T^, showing shared and unshared orthologous genes clusters

The pan-genome analysis of strains *P. alpniumensis* MSAK28401^T^, *P. citreus* DSM 20549^T^, and *P. rifietoensis* M8^T^ was depicted in a Venn diagram (Fig. 6). The three strains of *Planococcus* possessed 3363 gene families, whereas a “core” genome comprised 2853 clusters of orthologous, accounting for 84.5% of all gene families. Most of the annotation functions of homologous clusters were involved in biological process, hydrolase activity, ion binding, molecular function, and transferase activity. A total of 63 unshared protein clusters were found in the strain MSAK28401^T^, whereas 6 and 4 unshared protein clusters were found in the strains of *P. citreus* DSM 20549^T^ and *P. rifietoensis* M8^T^, respectively. Remarkably, the number of unshared clusters of the strain MSAK28401^T^ was higher than those of these related species. Approximately 84% of unshared clusters involved biological processes, such as those involving nucleobase-containing compounds, cellular aromatic compounds, macromolecules, nitrogen compounds, and heterocycle metabolism, thereby indicating the unique advantages in its biological process compared with the other related strains.

### Secondary metabolites

**Fig. 7:**
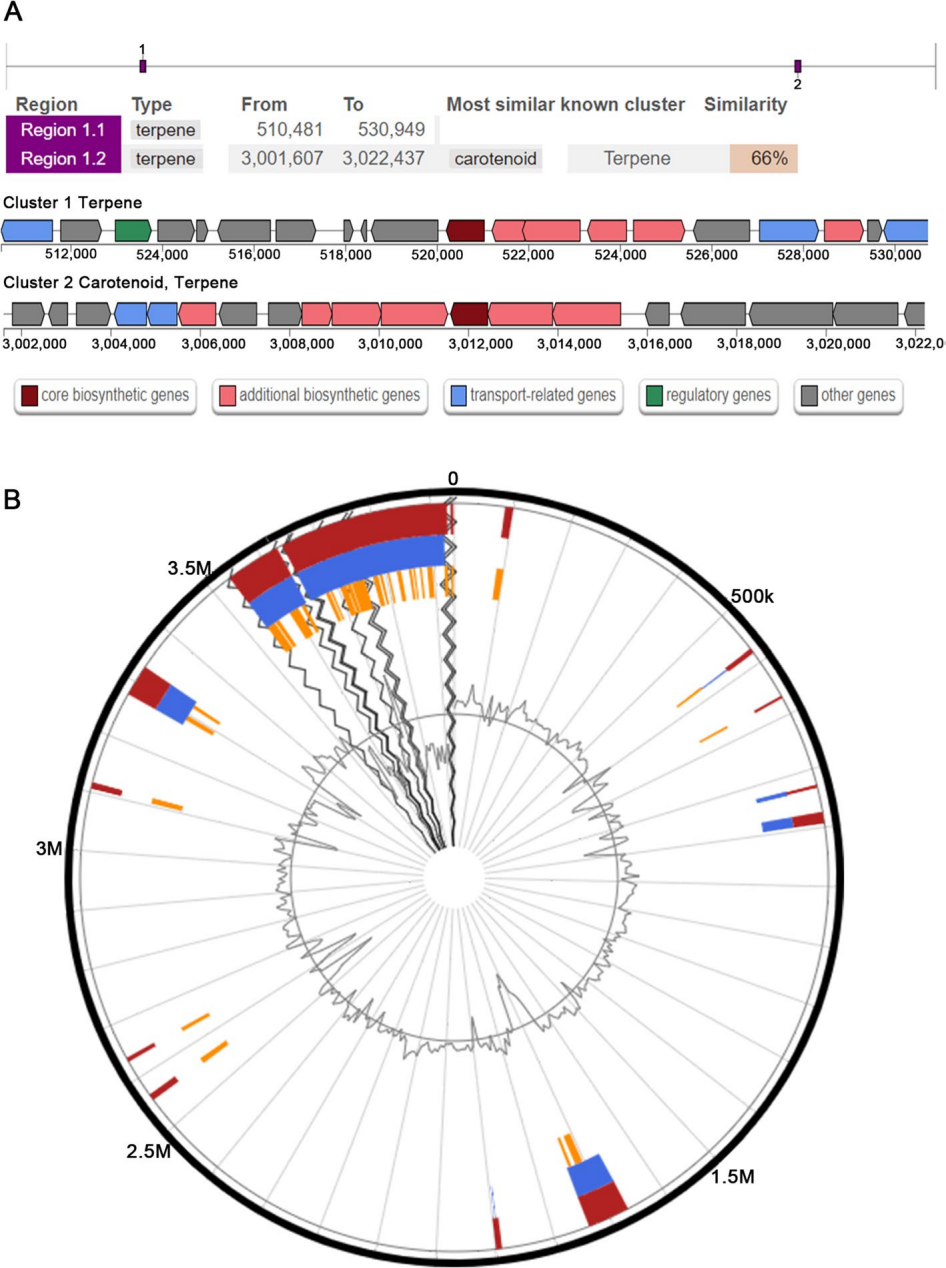
Secondary metabolism and genomic islands analysis in MSAK28401^T^ genome. **a** antiSMASH predicted biosynthetic gene clusters. **b** The predicts Genomic Islands (GIs) of the strain MSAK28401^T^. The red represents the prediction by integrated approach; blue displays results via IslandPath-DIMOB; orange represents genomic islands predicted using SIGI-HMM.

Screening the genes of secondary metabolites showed two different genes clusters, which both belong to the terpene biosynthesis-related clusters (Fig. 7A). Cluster 1 displayed orphan Biosynthetic gene clusters (BGCs), which were unable to identify the known homologous gene cluster. Cluster 2 (3,001,607-3,022,437 nucleotides) was 66% similar to the known BGC (BGC0000645), which was a gene cluster comprising carotenoids biosynthetic carotenoids. Nevertheless, the low similarity of predicted gene clusters may represent the production of new metabolites.

### Islands of genome

Thirty-seven genomic islands were predicted in this new strain MSAK28401^T^ by IslandViewer 4, and the localization of the predicted genomic islands is shown in Fig. 7B. The 37 genomic islands were made up of 971 genes from the range of 4000–320,000 bp. Among these, 581 genes were hypothetical proteins with no function, 29 genes were mobile element protein, but genes producing secondary metabolites were not found within the genomic islands.

## Discussion

The species of *Planococcus* are the dominant species in many marine environments, e.g., deep sea, salt marshes, and intertidal zones [5]. These aerobic heterotrophic bacteria degrade a variety of hydrocarbons, so they can make a significant contribution to the reduction of hydrocarbon contamination in the marine environment [5, 15]*.* Thirty species of *Planococcus* have been characterized. Notably, six typical strains have been found in the Antarctic, namely, *P. faecalis* [16], *P. versutus* [17], *P. maitriensis* [18], *P. antarcticus, P. psychrophilus* [9], and *P. mcmeekinii* [19]. In this work, we isolated and identified a new species strain MSAK28401^T^ belonging to the genus *Planococcus* from Antarctic krill.

Defining a new species involves two consecutive steps, namely, 16S rRNA gene analysis and calculation of several parameters of the genome [20]. In conformity to this scheme, we analyzed the 16S rRNA sequence of strain MSAK28401^T^ and found that the similarity between the corresponding gene sequence and related stains within genus *Planococcus* was less than 98.7%. This finding supported the idea that this strain might be a new species, because some species recently proposed in the genus *Planococcus* had similar or highly similar values in the 16S rRNA gene [17, 21, 22]. Chun et al. proposed a minimum standard to the taxonomy of prokaryotes using genomic data [20]. The whole-genome analysis results showed that the threshold values of ANI for species differentiation were 95–96%, which were generally accepted. The calculated ANI values of the genome of related strains of *Planococcus* were less than 91%, thereby indicating that the strain belongs to a novel species within the genus *Planococcus*. Furthermore, the morphology, phenotype, and whole-genome analysis of the strain MSAK28401^T^ showed that it represented a new member of the genus *Planococcus* and was named *P. alpniumensis* sp. nov.

Genus *Planococcus* is a halophilic bacterium known for producing various secondary metabolites [23], which are often referred to as anti-inflammatory, antimicrobial, pharmaceutically significant, and chemotherapeutic [24]. Ganapathy et al. identified a new carotenoid (methyl glucosyl-3,4-dehydro-apo-8-lycopenoate) with antioxidant activity from *P. maritimus* MKU009 [25]. Nevertheless, two clusters of genes that may be involved in the synthesis of terpenes were discovered by scanning potential secondary metabolites in strain MSAK2840^T^. Cluster 2 had 66% similarity with the gene cluster of the carotenoid biosynthesis of *Halobacillus halophilus* DSM 2266, which can help the strain resist oxidative stress. Genes associated with aromatic compound metabolism, one of the most common and persistent contaminants in environments [26], were found. In general, degradation of hydrocarbons, e.g., salicylate, gentisate, and quinate degradation, was a function of *Planococcus* [23].

By identifying vertical genetic homologous gene clusters from unique common ancestors, comparative analysis can help clarify the relationship between different species and the evolution and adaptability of the genome [23, 27]. The strain MSAK28401^T^ shared 2853 gene clusters with *P. citreus* DSM 20549^T^ and *P. rifietoensis* M8^T^, and had 63 unshared protein clusters. The functional distribution of homologous gene families in core genomes showed that most homologous gene families encode the basal metabolism of bacteria, such as protein processing, folding, and secretion and DNA and RNA metabolism [28]. Notably, the number of unshared clusters in strain MSAK28401^T^ was significantly higher than these related species among themselves (Fig. 6). The biological processes of unshared clusters of strain MSAK28401^T^ are aromatic compound, nitrogen compound, macromolecule, and heterocycle metabolic processes, indicating the unique advantages in its biological process than other related strains.

## Conclusion

The analysis of genomic, chemotaxonomic, and phenotypic traits showed that the strain MSAK28401^T^ belongs to a new species of the genus *Planococcus*, named *P. alpniumensis* sp. nov, whose type strain is MSAK28401^T^. Furthermore, genomic characterization and comparative analysis showed that the strain *P. alpniumensis* MSAK28401^T^ contained many genes related to the metabolism and transportation of amino acids and carbohydrates, thereby suggesting that MSAK28401^T^ might possess n efficient nutrient uptake system. Screening the secondary metabolite genes found two different types of terpene biosynthesis-related clusters. Cluster 2 was similar to carotenoids (66% of genes showed similarity), thereby indicating that these predicted gene clusters may represent the production of new metabolites. Finally, genes (catechol 2,3-dioxygenase (gene 0402), 4-oxalocrotonate tautomerase (gene 2794), and S-(hydroxymethyl) glutathione dehydrogenase / alcohol dehydrogenase (gene 2852)) involved in the degradation of aromatic compounds (e.g., salicylate, gentisate, and quinate) were identified, indicating the potential metabolism of an aromatic compound of the new species.

### Description of *P. alpniumensis* sp. nov.

This study reported the genomic information and phenotypic characteristics of the new strain *P. alpniumensis* MSAK28401^T^ isolated from Antarctic krill. Cells were Gram-stain positive, aerobic, non-motile and coccoid (0.89–1.05 μm). After 3 days of culture on LB medium at 28 °C, the colonies were orange and round, no-flagellum, no-spore, no-mobility, growth temperature range from 4 to 45 °C, and 30 °C is the optimum growth temperature, as well as anaerobic growth does not occur. Tween 40 hydrolyzes the colony, while gelatin and casein do not. The carbon sources were _D_-turanose, 3-methyl glucose, _D-_galactose, _D_-sorbitol, _D_-mannitol, _D_-mannose, _D_-lactic acid methyl ester, and _D_-gluconic acid, but not dextrin, *β*-methyl-_D_-glucoside, *α*-_D_-lactose, _L_-pyroglutamic acid, _D_-lellobiose, _D_-melibiose, _D_-glucose, _L_-aspartic acid, _D_-glucose-6-PO4, _D_-fructose, _D_-arabitol, _L_-glutamic acid, _D_-galacturonic acid, _L_-malic acid, _L_-alanine, _L_-arginine, _D_-aspartic acid, _D_-glucuronic acid, _L_-galactonic acid lactone, glucuronamide, and quinic acid. C_15:0_ anteiso, C_16:1_ ω7c alcohol, C_15:0_ iso, C_16:0_ iso, C_17:0_ anteiso, and C_14:0_ iso were the major fatty acids (> 5%) of the strain. Content of DNA G + C was 47.15%. The type species was *P. alpniumensis*, MSAK28401^T^ (KCTC 43283^T^ and MCCC 1k05448^T^).

## Methods

### Bacterial isolation

Antarctic krill was collected from Antarctica (58°33.1″ W, 63°6.3″ S) in 2016. It was washed with sterile seawater thrice under aseptic conditions to remove superficial residual sediments and microbes. Three Antarctic krill samples from a collected site were ground and homogenized as one specimen. Then, the milled samples were diluted with approximately 1 ml of sterile water, collected in a 2 ml aseptic centrifuge tube, and centrifuged at 3500 rpm for approximately 5–10 min. An inoculation loop was used to obtain a small amount of supernatant liquid, which was spread on agar-mixed LB. Bacteria in inoculated dishes were allowed to multiply at 10 °C until the colonies became visible. The colonies were randomly isolated from the agar plates, picked, and sub-cultured almost thrice under the same conditions. The same strains were preserved in 20% glycerin liquid medium at − 80 °C for future use.

### Phylogenetic tree construction

16S rRNA gene sequence of strain MSAK28401^T^ was amplified and sequenced through the sequencing DNA service of TSINGKE Biological Technology, China and then compared with the EzBioCloud database [29]. ClustalW program was used for sequencing against the closest type strains [30] to analyze phylogeny. The Neighbor-Joining phylogenetic tree was established using MEGA-X [31]. The robustness of the phylogenetic tree was evaluated through bootstrap analysis (1000 replicates) [32].

### Phenotypic characterization

After incubating the MSAK28401^T^ strain on LB-Agar-Powder plates for 48 h at 25 °C, transmission electron microscopy confirmed the morphological characteristics. Motility was examined by stab-culture in semi-solid medium according to the method of Gerhardt et al. Oxidase activity was tested using 1% (w/v) tetramethyl-p-phenylenediamine. Formation of spores was monitored by phase-contrast microscopy on cells cultured on LB agar at 30 °C for up to 7 days. Growth at different temperatures (4, 10, 16, 25, 30, 37, 45, and 50 °C) was determined and bacterial concentration was measured as optical density at 600 nm. Under manufacturer-indicated conditions, phenotypic characterization of this strain and two reference strains (*P. citreus* DSM20549^T^ and *P. rifietornsis* M8^T^, which were obtained from Marine Culture Collection of China, MCCC) were identified using Biolog Gen III microstation. Strains *P. citrus* DSM20549^T^, *P. rifietornsis* M8^T^, and the strain MSAK28401^T^ were incubated together at 25 °C for 30 h and the results were tested.

### Chemotaxonomic analysis

For cellular fatty acid analysis, strains MSAK28401^T^, *P. citreus* DSM20549^T^, and *P. rifietornsis* M8^T^ were incubated together on LB-Agar-Powder at 25 °C for 2 days. Culture was harvested and prepared, and fatty acid methyl esters were separated based on the method proposed by Sasser [33] and were tested by the MIDI Sherlock Microbial Identification system.

### Genome sequencing and mining

Total DNA of the genome was purified from a purely cultured strain MSAK28401^T^ using a DNA extraction kit (TaKaRa, Japan) following the manufacturer’s protocol. PacBio sequencing and analysis were conducted by OE Biotech Co., Ltd. (Shanghai, China). The total DNA obtained was subjected to quality control via agarose gel electrophoresis and quantified by Qubit. The library was constructed utilizing the SMRTbell template prep kit 1.0 from Pacific Biosciences. Single-molecule real-time (SMRT) sequencing was performed on the PacBio Sequel platform. SMRT Analysis 2.3.0 was used to filter low-quality reads [34, 35]. The filtered reads were assembled into a contig without gaps. Falcon was used for the de novo assembly of these reads [36]. This draft genome sequence of MSAK28401^T^ was collected in GenBank and was given the accession number JAAMTH000000000.

A circular genome map of this strain MSAK28401^T^ was generated with CGView server (http://cgview.ca/) [37, 38]. Gene prediction of the assembled genome was performed with Prodigal v2.6.3 [39], and an assembled genome was masked with RepeatMasker v4.0.7 [40]. Annotation was completed with Rapid Annotation using Subsystem Technology (RAST 2.0) [2, 41, 42]. The genome-BLAST Distance Phylogeny (GBDP) [43] method compared these whole-genome sequences at nucleotide level, calculates DNA-DNA hybridization (dDDH) value, and a phylogenetic tree was constructed. The Average Nucleotide Identity (ANIb) value between genomes was calculated using JSpeciesWS Online Service [44]. Predictive genes were functionally classified using e-values of 1e-5 in five databases, namely, Non-Redundant Protein Database (NR), Gene Ontology (GO) Database, Swiss-Prot, Clusters of Orthologous Groups (COG), and Kyoto Encyclopedia of Genes and Genomes (KEGG)(http://www.genome.jp/kegg/pathway.html) [45, 46].

### Pan-genome and comparative genome-wide analysis

To compare genomes, the reference genome sequence of this bacteria was downloaded from the GenBank database. The pan-genome sequence comparative analysis of this strain MSAK28401^T^ was performed using the GBDP method [43]. Genomic homogeneous clustering analysis, including the genetic ontogeny of all predicted protein-coding genes, was performed using OrthoVenn2 [47].

## Additional files

**Additional file 1.** The similarity of bacterial 16S rRNA.

**Additional file 2.** Subsystems of genes according to SEED database (RAST server).

**Additional file 3.** Carbohydrate-Active enZYmes (CAZy) database annotated classification statistics.

**Additional file 4.** KEGG database annotated classification statistics.

**Additional file 5.** KEGG database notes summary table.

## Data Availability

All data generated or analyzed during this study are included in this published article and its supplementary information files. The datasets presented in this study can be found in online repositories. The datasets generated and/or analyzed during the current study are available in the [NCBI] repository, [https://www.ncbi.nlm.nih.gov/] [JAAMTH000000000].

## Abbreviations

ANI: Average nucleotide identity
DDH: DNA-DNA hybridization
LB: Luria-Bertani
SMRT: Single-molecule real-time
GBDP: The Genome-Blast Distance Phylogeny
NR: Non-Redundant Protein Database
GO: Gene Ontology
COG: Clusters of Orthologous Groups
KEGG: Kyoto Encyclopedia of Genes and Genomes
RAST: Rapid Annotation using Subsystem Technology
GT: glycosyl transferases
GH: glycosyl hydrolases
BGCs: Biosynthetic gene clusters
