# Supplementary material for: Whole genome sequencing and comparative genomic analyses of Planococcus alpniumensis MSAK28401T, a new species isolated from Antarctic krill
Source: BMC Microbiol. 2021 Oct 22;21:288. doi: 10.1186/s12866-021-02347-3 (PMC8532331; doi:10.1186/s12866-021-02347-3)
Supplement: Supplementary file 1 — Additional file 1. The similarity of bacterial 16S rRNA. [file 12866_2021_2347_MOESM1_ESM.docx]

**Additional Files**

**Table S1** The similarity of bacterial 16S rRNA.

| **Name** | **strain** | **Accession** | **Similarity (%)** |
| --- | --- | --- | --- |
| *Planococcus citreus* | DSM 20549^T^ | RCCP01000013 | 98.62 |
| *Planococcus rifietoensis* | M8^T^ | CP013659 | 98.55 |
| *Planococcus maitriensis* | S1^T^ | AJ544622 | 98.43 |
| *Planococcus dechangensis* | NEAU-ST10-9^T^ | JQ762282 | 98.20 |
| *Planococcus maritimus* | DSM 17275^T^ | CP016538 | 97.79 |
