# Supplementary material for: Whole genome sequencing and comparative genomic analyses of Planococcus alpniumensis MSAK28401T, a new species isolated from Antarctic krill
Source: BMC Microbiol. 2021 Oct 22;21:288. doi: 10.1186/s12866-021-02347-3 (PMC8532331; doi:10.1186/s12866-021-02347-3)
Supplement: Supplementary file 2 — Additional file 2. Subsystems of genes according to SEED database (RAST server). [file 12866_2021_2347_MOESM2_ESM.docx]

**Additional Files**

**Table S2** Subsystems of genes according to SEED database (RAST server).

| **Subsystems** | **Number of genes** |
| --- | --- |
| Cofactors, Vitamins, Prosthetic Groups Pigments | 138 |
| Cell Wall and Capsule | 56 |
| Virulence, Disease and Defense | 58 |
| Potassium metabolism | 9 |
| Miscellaneous | 28 |
| Phages, Prophages, Transposable elements, Plasmids | 4 |
| Membrane Transport | 56 |
| Iron acquisition and metabolism | 18 |
| RNA Metabolism | 50 |
| Nucleosides and Nucleotides | 103 |
| Protein Metabolism | 203 |
| Cell Division and Cell Cycle | 3 |
| Motility and Chemotaxis | 7 |
| Regulation and Cell signaling | 22 |
| DNA Metabolism | 72 |
| Fatty Acids, Lipids, and Isoprenoids | 74 |
| Nitrogen Metabolism | 9 |
| Dormancy and Sporulation | 9 |
| Respiration | 43 |
| Stress Response | 59 |
| Metabolism of Aromatic Compounds | 7 |
| Amino Acids and Derivatives | 266 |
| Sulfur Metabolism | 8 |
| Phosphorus Metabolism | 25 |
| Carbohydrates | 214 |
