# Supplementary material for: Whole genome sequencing and comparative genomic analyses of Planococcus alpniumensis MSAK28401T, a new species isolated from Antarctic krill
Source: BMC Microbiol. 2021 Oct 22;21:288. doi: 10.1186/s12866-021-02347-3 (PMC8532331; doi:10.1186/s12866-021-02347-3)
Supplement: Supplementary file 3 — Additional file 3. Carbohydrate-Active enZYmes (CAZy) database annotated classification statistics. [file 12866_2021_2347_MOESM3_ESM.docx]

**Additional Files**

**Table S3** Carbohydrate-Active enZYmes (CAZy) database annotated classification statistics.

| **CAZyme type** | **Number of genes** |
| --- | --- |
| Glycosil Hydrolases (GH) | 21 |
| Glycosil Transferases (GT) | 24 |
| Carbohydrate-binding Module (CBM) | 5 |
| Carbohydrate Esterase (CE) | 18 |
| Polysaccharide Lyase (PL) | 1 |
| **Total** | 79 |
