# Supplementary material for: Whole genome sequencing and comparative genomic analyses of Planococcus alpniumensis MSAK28401T, a new species isolated from Antarctic krill
Source: BMC Microbiol. 2021 Oct 22;21:288. doi: 10.1186/s12866-021-02347-3 (PMC8532331; doi:10.1186/s12866-021-02347-3)
Supplement: Supplementary file 4 — Additional file 4. KEGG database annotated classification statistics. [file 12866_2021_2347_MOESM4_ESM.docx]

**Additional Files**

**Table S4** KEGG database annotated classification statistics.

| **Pathway modules** | **Total** | **Number of genes related to** | | |
| --- | --- | --- | --- | --- |
|  |  | **Complete pathways** | | **Incomplete pathways** |
| **Carbohydrate metabolism** |  | |  |  |
| Central carbohydrate metabolism | 82 | | 66 | 16 |
| Other carbohydrate metabolism | 49 | | 20 | 29 |
| **Energy metabolism** | | | | |
| Carbon fixation | 51 | | 0 | 51 |
| Methane metabolism | 12 | | 4 | 8 |
| ATP synthesis | 21 | | 11 | 10 |
| **Lipid metabolism** | | | | |
| Fatty acid metabolism | 23 | | 12 | 11 |
| Lipid metabolism | 8 | | 4 | 4 |
| **Nucleotide metabolism** | | | | |
| Purine metabolism | 22 | | 22 | 0 |
| Pyrimidine metabolism | 15 | | 3 | 12 |
| **Amino acid metabolism** | | | | |
| Serine and threonine metabolism | 9 | | 5 | 4 |
| Cysteine and methionine metabolism | 25 | | 17 | 8 |
| Branched-chain amino acid metabolism | 23 | | 15 | 20 |
| Lysine metabolism | 29 | | 9 | 15 |
| Arginine and proline metabolism | 12 | | 6 | 6 |
| Polyamine biosynthesis | 6 | | 4 | 2 |
| Histidine metabolism | 10 | | 10 | 0 |
| Aromatic amino acid metabolism | 23 | | 14 | 9 |
| Other amino acid metabolism | 1 | | 0 | 1 |
| **Glycan metabolism** | | | | |
| Glycosaminoglycan metabolism | 1 | | 0 | 1 |
| **Metabolism of cofactors and vitamins** | | | | |
| Cofactor and vitamin metabolism | 124 | | 61 | 63 |
| **Biosynthesis of terpenoids and polyketides** | | | | |
| Terpenoid backbone biosynthesis | 18 | | 10 | 8 |
| Plant terpenoid biosynthesis | 1 | | 0 | 1 |
| **Biosynthesis of other secondary metabolites** | | | | |
| Biosynthesis of phytochemical compounds | 1 | | 0 | 1 |
| Biosynthesis of other antibiotics | 1 | | 0 | 1 |
| **Xenobiotics biodegradation** | | | | |
| Aromatics degradation | 3 | | 0 | 3 |
| **Others** | | | | |
| Drug resistance | 5 | | 0 | 5 |
